# Optimizing the Properties of Hybrids Based on Graphene Oxide for Carbon Dioxide Capture

**DOI:** 10.1021/acs.iecr.1c02922

**Published:** 2022-01-13

**Authors:** Yating Ye, L. Vega Martín, M. J. Sánchez Montero, D. López-Díaz, M. M. Velázquez, M. D. Merchán

**Affiliations:** † Departamento de Química Física, Facultad de Ciencias Químicas, Universidad de Salamanca, E-37008 Salamanca, Spain; ‡ Grupo de Nanotecnología, Universidad de Salamanca, E37008 Salamanca, Spain; § Laboratorio de Nanoelectrónica y Nanomateriales, USAL-NANOLAB, Universidad de Salamanca, E37008 Salamanca, Spain; ∥ Departamento de Química Analítica, Química Física e Ingeniería Química, Universidad de Alcalá. 28871 Alcalá de Henares, Madrid, Spain

## Abstract

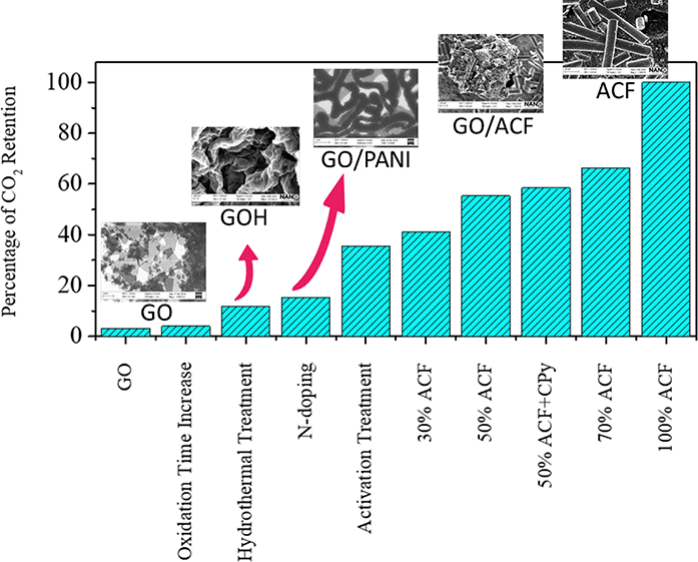

The reduction of CO_2_ emissions and its elimination from the atmosphere has become one of the major problems worldwide, since CO_2_ is the main cause of the greenhouse effect and climate change. In recent years, a great number of carbonaceous materials that can be used as CO_2_ adsorbents have been synthesized. The strategy is usually to synthesize the materials and determine their adsorption capacity without studying previously the factors that influence this capacity. In this work, different properties of the adsorbents are analyzed to study their influence on the CO_2_ adsorption capacity. For this purpose, 10 adsorbents have been synthesized using different strategies and characterized with X-ray photoelectron spectroscopy, X-ray diffraction, and micro-Raman spectroscopy. The percentage of sp^2^ carbons, the position of the D + D′ peak of the second-order Raman spectrum, the micropore volume, and the grain size of the C sp^2^ domains have been related to the amount of CO_2_ adsorbed by the adsorbents. The results confirm a linear relationship between the volume of the micropores and the CO_2_ uptake and it proves that CO_2_ retention is favored in those materials that, in addition to having a high volume of micropores, also have low grain size of C.

## Introduction

Multiple human activities and their negative impact on the environment have led researchers to dedicate a high number of working hours to retaining and eliminating CO_2_, which is one of the main causes of the greenhouse effect.^1,2^ The most promising and effective technologies have been adsorption^3^ and absorption;^4,5^ however, because of the disadvantages of absorption on liquids such as corrosion of the equipment and the high energy cost of regeneration,^6,7^ adsorption on solids for capture and storage has made it the most efficient and economical technology. Researchers have focused their attention on solid-based adsorbents that are easy to handle, ecofriendly, economical, and easy to regenerate.^8^ There is no lack of papers in the literature dedicated to improving the CO_2_ retention capacity of adsorbents.^9^ However, the large-scale production of nanomaterials for CO_2_ capture application still needs more work.^10,11^

According to recent published reviews regarding CO_2_ retention,^3,9,12^ the main adsorbents studied are activated carbon,^13,14^ porous carbon fibers,^15,16^ graphene derivatives,^17−19^ and noncarbonaceous adsorbents such as metallic organic frameworks (MOFs),^20−22^ zeolites,^23^ porous polymers,^24^ materials based on alkali metals and materials based on metal oxides.^25^ Carbon-based adsorbents such as activated carbons,^13,14^ fibers, and nanotubes,^15,16^ carbon microsheets,^17^ graphite, or graphene and its derivatives^18,19^ are identified as promising adsorbents, because of their high availability, economic profitablility, high specific surface area, ease of modifying the structure of the pores, and the possibility of functionalizing the surface.^26^ The other two solid materials most considered as efficient CO_2_ adsorbents are zeolites and MOFs, because of their high porosity and specific nature.^3,9^

In 2020, Hao et al. prepared a composite material of carbon nanotubes with numerous heteroatoms that gave one of the best values for adsorption capacity, viz., a maximum of 5.7 and 3.7 mmol/g of CO_2_ adsorbed at 273 and 298 K, respectively.^27^ The increase in CO_2_ adsorption capacity is not only due to the improvement of the surface, but also the development of the 3D structure, which could accelerate the diffusion of the gas and maximize the filling of the micropores. Xu et al.^28^ presented a hybrid material prepared with MOFs and graphene oxide exhibiting the highest CO_2_ capture at 273 K reported to date. The MOF was modified using graphene oxide and a double salt of Zn and Cu, resulting in a solid with a high specific surface area and pore volume (1554 m^2^ g^–1^ and 0.711 cm^3^ g^–1^), which could maximize the yield increasing the CO_2_ adsorption capacity from 6.85 mmol g^–1^ to 9.02 mmol g^–1^, and the CO_2_/N_2_ selectivity at 1 bar increased 1.8 times that of pristine material.^28^ Chowdhury and Balasubramanian^29^ and Shang et al.^30^ both have studied the CO_2_ uptake on 3D materials from graphene. Chowdhury et al. proposed a physical modification at various temperatures to form a 3D rGO. On the other hand, Shang et al. proposed a chemical modification method to obtain composites. Both obtained a good surface area and pore volume; however, the CO_2_ uptake at 298 K was very different, viz., 2.45 and 8.02 mmol g^–1^ respectively.

In designing an ideal adsorbent material, particularly for CO_2_ adsorption, it is necessary to know the relationships between the material’s structure and its adsorption properties. The work of Firdaus et al.^31^ includes a comparative study of the capture of CO_2_ performed on graphene, carbon nanotubes, zeolites, and metal-organic frameworks (MOFs) over the last 12 years. It is observed that the best yields for the capture of CO_2_ does not simply correspond to nanomaterials with the largest surface area or the largest pore volume. The presence of polar functional groups (epoxy, hydroxyl, carboxylic) in graphene derivatives, or the addition of metals to MOFs, may increase dispersion forces and therefore adsorption capacity.^31^ In addition to these two factors, surface area and pore volume, pore size could play an important role in improving CO_2_ uptake, and a strategy to improve it could focus on the design of materials with abundant and narrow micropores. According to a purely molecular sieving mechanism driven by gas diffusion, a reduction in pore size could lead to an improvement in CO_2_ selectivity.^31^ Normally, noncarbonaceous materials such as MOFs and zeolites contain an ordered internal pore or channel structure that leads to an increase in pore size and a decrease of CO_2_ capture at low pressure. Carbonaceous materials such as graphene derivatives or nanotubes contain heterogeneous slit-shaped holes and generally do not have a homogeneous pore network, so CO_2_ adsorption is more difficult to predict.^22^ In view of all these cases, it is reasonable to conclude that nanomaterials with high specific surface area, large pore volume, and relatively small pore size would provide ideal texture properties for the CO_2_ adsorbent, although there is a need for further investigation of the properties that govern the mechanisms that optimize this adsorption capacity.

The preparation of adsorbent materials based on carbon continues to be a promising strategy, because of its low cost, availability, possibility of modifying the porous structure, or even chemically functionalizing its surface.^9^ Therefore, in a previous work, we have prepared different hybrids based on graphene oxide using polyaniline and magnetite nanoparticles as the second components.^32^ Our results showed that these hybrids increase the CO_2_ retention more than 10 times, with respect to pure graphene oxides, and they showed that the CO_2_ uptake linearly increases with the micropore volume of solids. Accordingly, to develop new and high-quality adsorbents, it is necessary to understand which of the structural characteristics have a greater influence on the microporosity of these nanomaterials. With this objective in mind, in the current work, we have obtained graphene oxide-based nanomaterials prepared by different methodologies, which allow us to obtain materials with different structural properties, such as C sp^2^ percentage, grain size, or surface area.

Based on our previous experience,^32^ we have selected two types of graphene-oxide based materials: a graphene oxide doped with polyaniline, a strategy with which it was possible to increase the CO_2_ retention capacity of the starting graphene oxide more than 10 times,^32^ and hybrid hydrogels of graphene oxide with activated carbon microporous fiber (ACF) prepared by the hydrothermal method with different GO/ACF ratio.

## Experimental Section

### Chemical and Materials

Graphene oxide was synthesized by the oxidation of natural graphite flakes (99.02%) from Qingdao Super Graphite Co., Ltd.

The reagents used for graphite oxidation and at the activation process were NaNO_3_ (99%), H_2_SO_4_ (98% w/w), KMnO_4_ (>99%), H_2_O_2_ (30% w/w), KOH, HCl (35%), and aniline, which were provided by Sigma–Aldrich (St. Louis, MO). FeCl_3_·6H_2_O was supplied by Panreac Química SLU (Barcelona, Spain). All reagents were used without purification. We used ultrapure water from a RiOs and Milli-Q combined system from Millipore.

A commercial activated carbon fiber (ACF) supplied by Kynol Europe (Hamburg, Germany) was used. According to the details provided by the supplier, ACF was from Novoloid textile fiber and activated in a one-step process combining carbonization and activation at 900–1000 °C.

Carbon dioxide (CO_2_), nitrogen (N_2_), and helium (He) were supplied by Praxair, Inc., and Oxygen (O_2_) by L’Air Liquide, Madrid, Spain. The minimum purity was 99.999%.

### Synthesis and Activation of Polyaniline–Graphene Oxide Nanocomposite

Graphene oxide was obtained by oxidizing graphite flakes with a modified Hummer’s method proposed by our group,^33−35^ using 12 or 24 h of oxidation, respectively. The solids obtained are named GO12 and GO24, respectively.

The GO/polyaniline nanocomposite was synthesized via an in situ polymerization procedure previously reported.^36,37^ Briefly, aniline was added to graphene oxide (GO12) dispersions of 2 mg/mL prepared by sonication 1 h in 15 min cycles. The GO:aniline mass ratio was (15:85). The graphene oxide dispersions were mixed with aniline and sonicated for 1 h (15 min cycles). The polymerization was performed under stirring by careful addition of 6 mL of H_2_O_2_ (30%), 4.5 mL of HCl (37%) and 1 mL of FeCl_3_·6H_2_O (0.1 M). More details can be obtained from a previous work.^32^ Finally, the solids were washed with acetone to eliminate the excess of graphene oxide and dried under vacuum at 60 °C for 24 h and thermally activated at 400 °C under inert gas in a tubular furnace for 1 h. The adsorbent obtained is named GO12PANI.

Chemical activation of the nanocomposite was performed by dissolving 400 mg of the nancomposite in 20 mL of 7 M KOH stirring for 4 h at 400 rpm and 20 h of static contact.^38^ After that, the solid was filtered through a polycarbonate membrane (0.2 μm) and dried at 60 °C for 24 h. The next step of thermal activation was programmed at 550 °C under inert gas in a tubular furnace for 2 h by ramping the temperature at a rate of 2 °C/min. After activation, the product was washed with 0.1 M HCl solution and then dried under vacuum at 60 °C for 24 h. The solid obtained was named GO12PANIK.

### Synthesis of Hybrid Hydrogels from Graphene Oxide and Activated Carbon Fibers

The preparation of the GO/activated carbon fibers hydrogels was performed by introducing a mixture of the two components GO24 and activated carbon fibers (ACF) into a 100 mL Teflon-lined autoclave, so that the final GO24 concentration was always 2 mg/mL. For this, a 50 mL aqueous suspension of 4 mg/mL of GO24 homogenized in an ultrasound bath for 5 min was mixed with 50 mL of an aqueous suspension of ACF, and then ground and dispersed in an ultrasonic treatment for 5 min. To facilitate the grinding of the ACF to powder, a piece of cloth was dipped into liquid N_2_ and ground on an agate mortar. The mixture was stirred for 5 min in an ultrasound bath. Three samples were prepared in which the GO24/ACF weight ratio was 30/70, 50/50, and 70/30.

The Teflon-lined autoclave reactor was filled with 60 mL of the mixture and heated to 180 °C by ramping the temperature at a rate of 2 °C/min. The temperature was maintained for 12 h. The samples obtained were named GO24FH37, GO24FH55, and GO24FH73, respectively. After 12 h, the Teflon-lined autoclave was naturally cooled to room temperature and the product was filtered under vacuum over a 0.22 μm PVDF membrane. Finally, the samples were dried in an oven at 60 °C for 24 h. To favor the self-assembly between ACF and the GO, the sample containing GO24/ACF at a weight ratio of 50/50 was prepared by replacing the water used to prepare the suspension of the ACF with a 2.9 × 10^–4^ M (below cmc) solution of cetylpyridinium chloride GOFH55CPy. As a control, the GO24H sample was also prepared by introducing 60 mL of a 2 mg/mL aqueous suspension of GO24 into the hydrothermal reactor and subjecting it to the same treatment.

### Structural Characterization

X-ray photoelectron spectra (XPS) of powder samples were recorded in a PHI Versa Probe II (Physical Electronics, USA), equipped with an excitation source of Al Kα (1486.6 eV) at 25 W and a 1.3 V and 20.0 μA neutralizer. The high-resolution spectra were recorded working at an analyzer pass energy of 29.35 eV.

Powder XRD patterns were recorded in a Bruker D8 Advance powder diffractometer using Cu Kα_1,2_ radiation (λ = 1.54050 Å) between 5° and 80° (2θ) with a step size of 0.05° and a step time of 2.6 s. The tube operated at 40 kV and 30 mA. Interlayer spacing *d*_002_, values were obtained by Bragg’s law for (002) reflection. The grain size (*C*) was calculated from the (100) reflection, using the equation of Scherrer:^39^*C* = 0.9 λ/(β cos θ), where β is the half weight width (in radians) and θ is the diffraction angle.

The porous structure was determined by the 77 K N_2_ adsorption/desorption isotherms, over the relative pressure range of 0 to 1, in an ASAP 2010 (Micromeritics) system after degasification in 0.1 mbar and at 423 K for at least 12 h. The surface area was calculated with the BET equation within the range of relative pressure of 0.05 < *P*/*P*_0_ < 0.30.

The adsorption capacities of GO/ACF hydrogels and of GO24PANIK were evaluated by performing the CO_2_ adsorption isotherms at 273 K, over a relative pressure range of 0 to 0.034, in an ASAP 2010 (Micromeritics). The narrow microporosity was evaluated by the CO_2_ adsorption isotherms at 273 K in the ASAP 2010 (Micromeritics). The micropore volume (*V*_mp_) and the characteristic energy of adsorption (*E*_0_) were calculated using the Dubinin–Radushkevich model (see details in Section S2 of the Supporting Information) at relative pressures of <0.01.^40,41^

## Results and Discussion

As a part of our two strategies, we have selected two graphene oxides obtained from the exfoliation of graphite by chemical oxidation. Since the final characteristics of the material are dependent widely on the degree of oxidation,^33−35,42,43^ we have used graphene oxides obtained after different oxidation times and reduced via the hydrothermal method. Hydrothermal reduction is widely used because it is an environmentally friendly method that decreases the oxygen content cost effectively.^44−46^ As a third strategy, thermal^47^ and chemical activation with KOH^48,49,47^ has been tested, starting from a graphene oxide hybridized with polyaniline, since this provided good results as a CO_2_ adsorbent in a previous work.^32^ Finally, we have selected the combination of GO with activated carbon fibers, because fibers present a high degree of microporosity, and because the hybrids can increase the mechanical properties of fibers avoiding the necessity of handling the fibers in the form of finely divided powder.

### Characterization of Hybrids by XPS

Using X-ray photoelectron spectroscopy (XPS), the content of C, N, and O, the degree of oxidation, and reduction of the different samples, as well as the C sp^2^/C sp^3^ and C/O ratio, have been determined.

**Figure 1:**
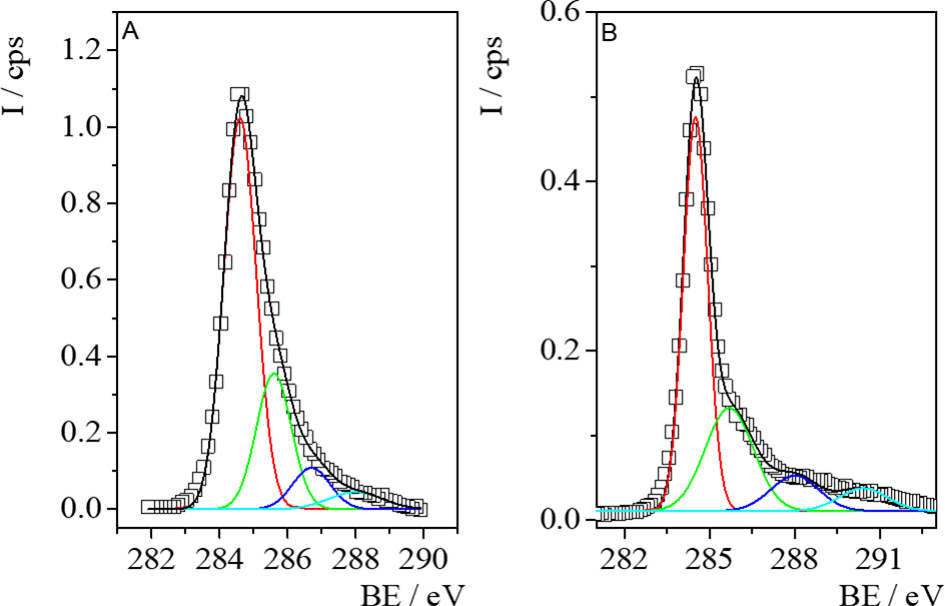
X-ray photoelectron spectra of C 1s core level of (A) N-doped composite GO12PANIK and (B) GO24/ACF composite (GO24FH37).

Wide spectra in the binding energy range 298–525 eV were obtained to identify the surface elements presented and to obtain a quantitative analysis (see Table S1 in the Supporting Information). Figure 1A shows the spectra for the C 1s core level, of GO12PANIK and an illustrative spectrum of a composite of GO with ACF, GO24FH37, is presented in Figure 1B. The other spectra are collected in Figures S1 and S2 in the Supporting Information. C 1s core-level spectra of GO12, GO24, and GOH are asymmetric lines that can be deconvolved into three functions assigned to aromatic carbon bonds (284.8 eV), to C–O bonds corresponding to alcohol or epoxy groups (286.4 eV) and to COO^–^ groups (287.9 eV).^50^^35,51^ Results in Table 1 show the three components of C 1s core level spectra for neat graphene oxides centered at 284.8, 286.4, and 288 eV, respectively. C 1s core-level spectra for N-doped GO composite (GO12PANI and GO12PANIK) should be fit to four peaks, a fourth peak centered at 285.6 eV assigned to the C–N bond appears.^52^ The appearance of the N 1s spectra between 412 and 490 eV (Figure S1 in the Supporting Information) and the peak of C–N bonds in C 1s core level at GO12PANI and GO12PANIK spectra unequivocally demonstrate the formation of the hybrid material between graphene oxide and PANI. The percentage of both C–O and COOH groups in the GO/PANI nanocomposites decreases, compared with that of graphene oxide. This fact shows that the interactions between graphene oxide and PANI is through those O groups. Simultaneously, the percentage of C sp^2^ increases in the composites with PANI due to the aromatic groups of PANI. This proves the functionalization of graphene oxide with the polymer PANI. Table 1 collects the binding energy values of each chemical group. The percentage of each chemical group is calculated from the area of each peak related to the total area of the band. For comparison, data corresponding to GO12PANI^32^ are also given in Table 1.

**Table 1:** Values of Binding Energies and Percentages of Different Groups for Nanocomposites and Hydrogels Obtained from XPS Measurements

sample	C 1s emission	max. binding energy (eV)	composition (%)	C/O	C sp^2^/C sp^3^
**GO12**	C=C	284.6	44 ± 3	2.3	0.8
	C–O	286.1	38 ± 2		
	COOH	287.9	18 ± 1		
					
**GO12PANI**^a^	C=C	284.6	64 ± 4	14.3	1.8
	C–N	285.6	20 ± 1		
	C–O	286.7	12 ± 1		
	COOH	288.0	4.0 ± 0.2		
					
**GO12PANIK**	C=C	284.6	63 ± 4	12.5	1.7
	C–N	285.6	27 ± 1		
	C–O	286.7	4.9 ± 0.5		
	COOH	288.0	5.1 ± 0.2		
					
**GO24**	C=C	284.8	60 ± 2	0.9	1.5
	C–O	286.4	28 ± 2		
	COOH	288.4	12 ± 1		
					
**GO24H**	C=C	284.8	51 ± 2	6.6	1.1
	C–O	286.4	40 ± 2		
	COOH	288.4	9 ± 1		
					
**GO24FH73**	C=C	284.5	53 ± 4	3.7	1.1
	C–O	285.7	28 ± 1		
	COOH ^–^	288.0	12 ± 1		
	CO_3_^–^	290.4	7.0 ± 0.2		
					
**GO24FH55**	C = C	284.5	55 ± 4	4.2	1.2
	C–O	286.0	23 ± 1		
	COOH	288	7 ± 1		
	CO_3_^–^	290.8	15.0 ± 0.2		
					
**GO24FH55CPy**	C = C	284.6	55 ± 4	4.1	1.2
	C–O	285.6	29 ± 1		
	COOH	288	7.3 ± 0.8		
	CO_3_^–^	290.8	8.7 ± 0.2		
					
**GO24FH37**	C = C	284.5	55 ± 4	4.5	1.2
	C–O	285.7	29 ± 1		
	COOH	288	9.8 ± 0.5		
	CO_3_^–^	290.4	6.2 ± 0.2		
					
**Carbon Fibers (ACF)**	C=C^–^	284.5	67 ± 4	10.3	2.1
	C–O	286.0	17 ± 1		
	COOH	288	9 ± 1		
	CO_3_^–^	290	7.0 ± 0.5		

a Data taken from ref (32).

C 1s core-level spectra for GO/ACF hydrogels in the 285 eV region are deconvolved into four surface functional group contributions with binding energies at 284.8, 286.4, 287.9, and 290 eV. The fourth component is carbonate (CO_3_^2–^) present in ACF.^53,54^Table 1 also shows that the hydrothermal treatment causes a decrease of the most oxidized groups attached at the edges of platelets, C=O groups, as expected since the hydrothermal process is a reduction process. It is interesting to notice that the hybrids containing ACF, present an almost constant percentage of C sp^2^. Besides, the percentage of C–O and C=O groups are almost independent of the ACF attached to the graphene oxide.

### XRD Analysis

**Figure 2:**
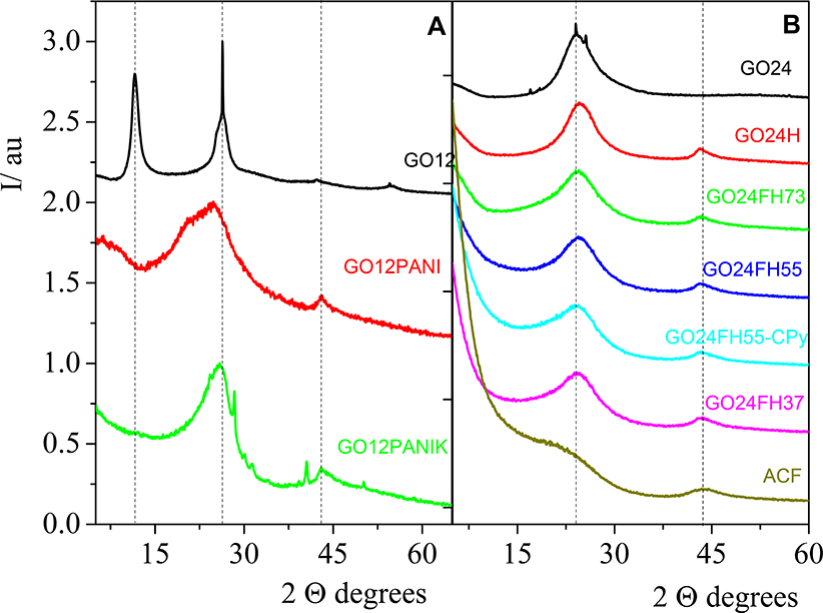
X-ray diffractograms of: (A) GO12, GO12PANI, and GO12PANIK; (B) GO24, GO24H, and GO24/ACF composites of different GO/ACF ratio. For the sake of clarity, the diffractograms are vertically shifted.

In samples obtained by different treatments, it is necessary to check if the treatments performed to the different composites modify the crystallinity and graphitization degrees of graphene oxide. Therefore, the XRD diffractograms and the Raman spectra of all samples were recorded. Concerning the XRD analysis, the diffractograms are plotted in Figure 2.

Previous studies have referred to the diffractogram of graphite, an intense crystalline peak at 2θ = 26.4° (lattice spacing calculated from the Bragg equation was 0.34 nm), assigned to the (002) diffraction peak.^46^ It is also referenced how after oxidation, the peak shifts to a lower angle at 2θ between 10° and 12° with a lattice spacing of 0.81 nm.^46^ The increase of the interlayer distance is due to the intercalation of water and oxygen functional groups on the basal plane.^46^ Besides, the peak disappears when graphene oxide is reduced by thermal treatment.^34^ In our samples, this peak only appears in GO12 (Figure 2A) prepared by 12 h of oxidation at 2θ = 11.6°. The lattice spacing calculated from the Bragg equation was 0.76 nm, in accordance with the value previously obtained by specular neutron reflectivity measurements for GO adsorbed at the air/water interface.^43,55^ In the case of samples obtained by the hydrothermal treatment, the disappearance of this peak can be attributed to the thermal reduction, while, in the case of GO24 prepared by oxidation during 24 h (Figure 2B), the absence of the peak can be attributed to the break of the *c*-axis order produced by the strong oxidation.^34,46^

In the XRD patterns of GO12/PANI composites (Figure 2A), we can observe two peaks at ∼25° and 43°, corresponding to the (002) and the (100) reflections, respectively, and the diffraction peak of graphene oxide disappears due to the exfoliation of GO during the polymerization of aniline.^56^ The interlayer distance value between carbon layers was calculated using Bragg′s law (*d*_002_ ≈ 0.36 nm). From the width at half height of (100) peak, and the Scherrer′s equation, we obtain the grain size, *C*, resulting in a value of ∼12 nm for GO12PANI and decreasing to ∼9 nm after chemical and thermal activation.

XRD patterns of the GO/ACF hydrogels present a broad (002) peak and a small peak from the (100) reflection. The hydrothermal reduction broadens and makes the (002) peak dominant,^46^ and the peak at 11° characteristic of GO is undetectable, Only in GO24FH37 with GO/ACF ratio 30/70 some reflection under 10° appears. The distance value between sp^2^ carbon layers *d*_002_ resulted around 0.36 nm for all the hydrogels. The grain size *C* is close to 5 nm for all the hydrogels (Table S2 in the Supporting Information). The decrease of the interlayer spacing from 0.76 nm for GO to 0.36 nm for hybrids is attributed to GO reduction due to thermal treatment. Thus, when the O-groups at the basal plane and at the edges decrease, the GO nanoplatelets are closer. A similar behavior has been observed for the grain size. ACF appears to be an amorphous material in view of the XRD diffractogram, so the formation of hybrids with ACF decreases the crystallinity, as previously justified by our group,^34^ and leads to lower grain size values (*C* ≈ 5 nm).

### Raman Analysis

**Figure 3:**
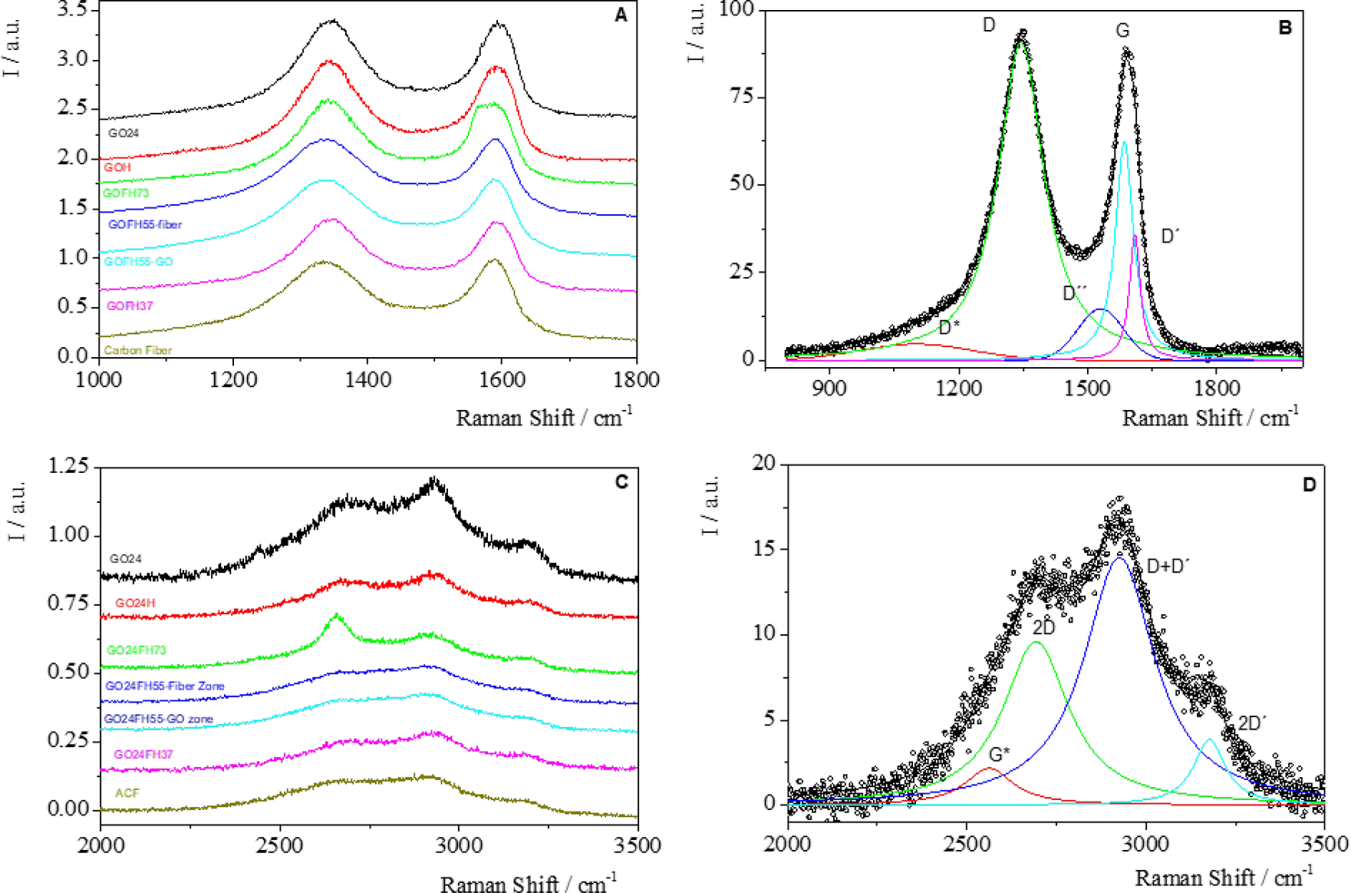
Raman spectra for hydrogels GO24/ACF: GO24, GO24H, GO24FH73, GO24FH55 at fiber zone, GO24FH55 at GO zone, GO24FH37 and carbon fibers: (A) first-order Raman spectra, (B) deconvolution of first-order Raman spectra of GO24FH37 hydrogel, (C) second-order Raman spectra, and (D) deconvolution of second-order Raman spectra for GO24FH37 hydrogel. For the sake of clarity, the spectra featured in panels (A) and (C) are vertically shifted.

The Raman spectra of the samples were recorded deposited onto silicon wafer. The analysis of the first- and second-order Raman spectra has made it possible to obtain information on the type of defects and vacancies on the C sp^2^ lattice^34,35,57^ generated in the carbon network during the preparation of hybrid materials. The spectra corresponding with the first-order Raman signal are plotted in Figure 3A. All samples present a Raman spectrum with the two bands D and G centered at ∼1350 and 1585 cm^–1^ respectively, characteristic of the graphenic materials. This reveals that the treatment used to fabricate the hydrogels retains the graphitization of graphene oxide. The G (∼1585 cm^–1^) peak is due to the bond stretching of sp^2^ carbons in rings and chains, while the D (∼1350 cm^–1^) peak originates from the breathing modes of the six membered rings that are activated by defects.^46^ All the hydrogels prepared with GO and ACF exhibit these characteristic peaks; even carbon fibers have a similar Raman spectrum.

The first-order Raman spectrum of carbonaceous materials cannot be simplified in two width bands (Figure 3A). According to our previous works,^34,35^ the Raman spectra of the first order can be fitted to five peaks (Figure 3B), assigned to D*, D, D″, G, and D′ bands. The D* band was previously observed in several carbonaceous materials^58,59^ and in the case of graphene oxide can be related to disordered graphitic lattices provided by the existence of Csp^3^ bonds. Besides, the D″ peak (∼1500–1550 cm^–1^) was related to the amorphous phases^60^ since its intensity decreased with the crystallinity. We also proved that the *I*_D_/*I*_G_ peak intensity ratio does not reveal significant differences about the number of defects on the samples. However, when the D′ band was interpreted according to the double-resonance mechanism, it can be related to the type of defects on the basal plane. Accordingly, *A*_D′_/*A*_D_ values close to 0.14 are characteristic of vacancies, while C sp^3^ and grain-boundary defects present values of 0.07 and 0.29, respectively.^61−63^Figure 3B shows an illustrative example of the deconvolution process corresponding to GOFH73. Similar behavior was observed for the rest of the samples. For details, all Raman spectra are collected at the Figure S3 in the Supporting Information and the best parameters obtained from fits are in Table S3 in the Supporting Information. In the case of PANI composites, the Raman spectrum of PANI completely masked the GO spectrum; therefore, it was not possible to analyze this.

**Figure 4:**
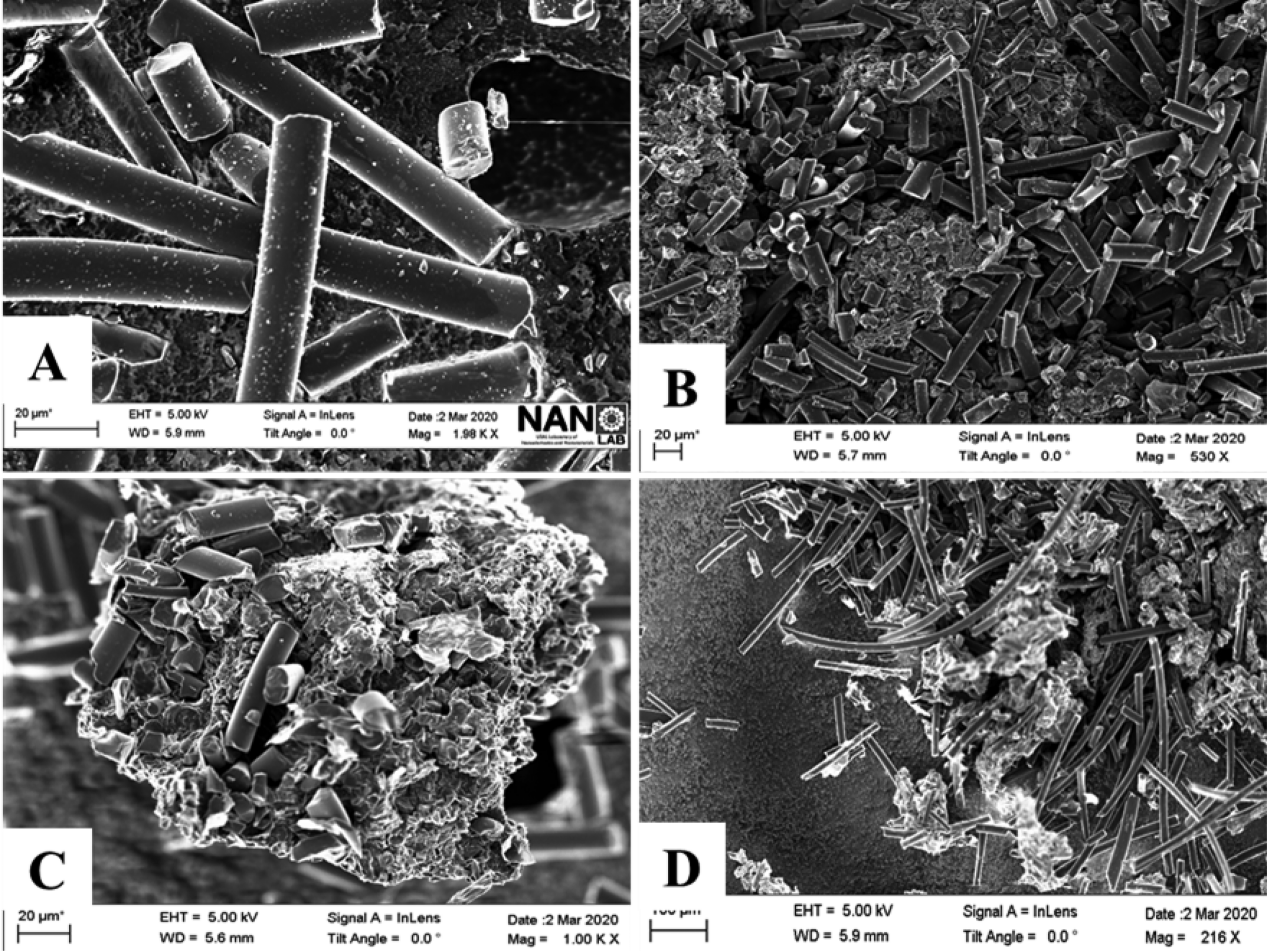
SEM images of mixed hydrogels of GO24 and carbon fibers: (A) activated carbon fibers, (B) GO24FH37, (C) GO24FH55, and (D) GOFH73.

To analyze the type of defects in each sample, we have calculated the ratio between the areas of the D′ and D bands (*A*_D′_/*A*_D_),^61−63^ and the values are shown in Table S4 in the Supporting Information. Results show that the *A*_D′_/*A*_D_ values for GO24 (0.11) and GO24H (0.10) are close to the value corresponding to vacancy defects and agree very well with the value previously obtained for graphene oxide synthesized by the oxidation of the same type of graphite.^35^ However, in the case of ACF, the *A*_D′_/*A*_D_ ratio was characteristic of sp^3^ defects (0.07). This type of defects was also observed in the hydrogels with different percentage of ACF when the Raman spectrum was taken in the fiber regions, however, the *A*_D′_/*A*_D_ values in the regions in which the GO sheets predominate are the same as the pure GO sheets (see Table S4). This behavior suggests that, within the hybrids, there are different domains in which fibers or GO sheets predominate. To confirm this fact, the SEM images of hybrids deposited onto silicon were taken and are shown in Figure 4.

As can be seen in Figure 4, different domains of both materials, ACF, and GO can be observed in the GO/ACF hydrogels. As was expected, when the carbon fibers percentage increases, the number of domains of fibers increases (see Figure 4D). This behavior is consistent with the Raman results in which a unique sample (for example, GOFH55 presents two different values of the *A*_D’_/*A*_D_ ratio (see Table S3 in the Supporting Information): 0.08 and 0.14, corresponding to sp^3^ defects and to vacancies defects, depending on the region selected to record the Raman spectrum, ACF, or GO, respectively.

The second-order Raman spectra of the hydrogels are plotted in Figure 3C. In a previous work, the spectra were fitted to four Lorentzian functions centered at ∼2500, 2690, 2930, and 3190 cm^–1^ previously assigned to G*-band interpreted according to the double resonance, because of an intervalley process involving an in-plane transverse optical (iTO) phonon and one longitudinal acoustic (LA) phonon,^64^ 2D (overtone), D+D′ (combination band), and 2D′ (overtone) band, respectively. The position of the combination band (D + D′) is related with the chemical composition in graphene oxide samples.^35^ In the next section, this parameter will be related with the CO_2_ adsorption capacity. Figure 3D shows an example of the Raman spectra of GOFH73. The experimental spectra agree very well with those calculated by the sum of the four Lorentzian functions from the best fit parameters shown in Table S5 in the Supporting Information.

### Porous Structure

The characterization of the texture of the solids, *S*_BET_ and volume of micropores has been performed using the adsorption isotherms of N_2_ at 77 K and of CO_2_ at 273 K. Figure S6 in the Supporting Information shows the N_2_ adsorption–desorption isotherms at 77 K for GO24/ACF hybrids and for ACF. According to Figure S6, ACF shows a type I isotherm, whereas all composites have a combination of type I and type IV isotherms. The small hysteresis loop suggests the existence of mesopores in composites. N_2_ isotherms for GO12/PANI hybrids could not be obtained completely because of the low N_2_ adsorption of the materials. Table 2 shows the surface area (*A*_BET_) values calculated from N_2_ adsorption isotherms and the BET equation. The micropore volume (*V*_mp_) and the characteristic energy of adsorption (*E*_0_) values were calculated from CO_2_ isotherms and the Dubinin–Radushkevich model.^32,40,65^ (See the details in Section S2 in the Supporting Information.)

**Table 2:** *A*_BET_ Obtained by N_2_ Adsorption Isotherms at 77 K^a^

sample	*A*_BET_ (m^2^/g)	*V*_mp_ (cm^3^/g)	*E*_0_ (kJ/mol)	*Q*_ads_ (mmol/g)
**GO12**	–	0.03	21.3	0.40
**GO12PANI**	–	0.07	23.3	0.82
**GO12PANIK**	–	0.15	28.2	1.93
**GO24**	–	0.01	16.8	0.22
**GO24H**	184	0.06	24.4	0.64
**GO24FH73**	562	0.15	22.1	2.22
**GO24FH55**	696	0.21	22.0	3.00
**GO24FH55CP**_**Y**_	704	0.22	21.8	3.17
**GOFH37**	815	0.24	21.8	3.59
**ACF**	1395	0.41	20.2	5.42

a Pore structure parameters obtained via the Dubinin–Radushkevich model from CO_2_ adsorption isotherms (273 K and *p*_CO_2__ = 1 bar) for GO/PANI composites and for GO/ACF hydrogels.

Table 2 clearly shows that graphene oxides (GO12 and GO24) present negligible values of the surface area (*A*_BET_) and volume of micropore (*V*_mp_), with anticipated low CO_2_ retention capacity. Although the *A*_BET_ and *V*_mp_ values obtained for GO24H are low, 184 m^2^/g and 0.057 cm^3^/g, respectively, it can be said that the hydrothermal treatment develops a certain degree of porosity. Doping with PANI does not increase *A*_BET_ determined by N_2_, although it develops some microporosity determined by CO_2_ adsorption at 273 K. Chemical activation of GO12PANI with KOH, followed by thermal activation at 550 °C, develops microporosity accessible to CO_2_ molecules, as observed by the *V*_mp_ value obtained for GO12PANIK. However, the activation treatment does not develop microporosity accessible to N_2_.

GO/ACF hydrogels show an increase of both *A*_BET_ and *V*_mp_ as the percentage of ACF increases, but they do not reach the values of the ACF. Since ACF is a highly microporous material, it gives the hydrogel very useful textural properties for CO_2_ retention. The low values found for *E*_0_, the characteristic energy value (16–28 kJ/mol) suggests a physisorption mechanism for all materials. *E*_0_ values vary as a function of the micropore size. The narrower micropores have higher *E*_0_ values. The microporosity developed in the GO12PANIK composite is very narrow. All the GO/ACF hydrogels have very similar *E*_0_ values, indicating that the micropores have the similar size.

### CO_2_ Adsorption on Composites

**Figure 5:**
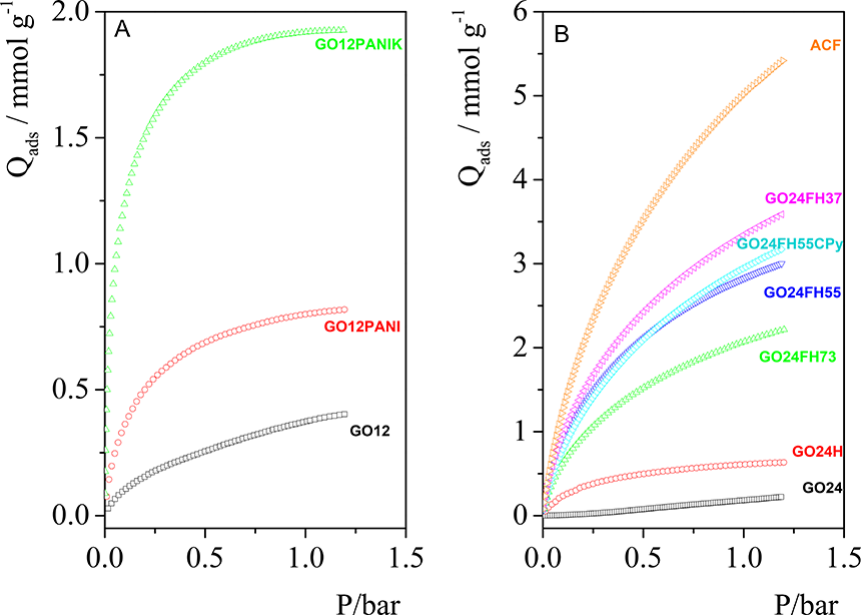
CO_2_ adsorption isotherms recorded at 273 K (A) for GO12 and composites GO12PANI and GO12PANIK and (B) for mixed hydrogels at different GO24/ACF ratios. GO isotherms were included as benchmarks.

As can be seen in Table 2, the CO_2_ uptake for the materials increases as follows: GO12 ≈ GO24 < G24OH < GO12PANI < GO12PANIK < GO24FH73 < GO24FH55 < GO24FH55CPy < GO24FH37 < ACF. GO12 and GO24 present very low CO_2_ adsorption capacity (∼0.40 and 0.22 mmol/g at 1 bar, respectively), which allows us to affirm that the oxidation time of graphene oxide is not a determining parameter in the CO_2_ retention capacity. When doping with polyaniline, GO12PANI achieved a CO_2_ uptake of 0.8 mmol/g at 1 bar, 2 times higher than that obtained for the original GO12. This behavior agrees with the results obtained previously.^32,65−71^ To improve the adsorption capacity of our materials, the N-doped sample is subjected to chemical activation treatment with KOH,^66,72^ followed by a thermal activation treatment at 550 °C. CO_2_ adsorption isotherms are shown in Figure 5. The result obtained, in terms of adsorption capacity for the composite activated, GO12PANIK, was 1.93 mmol/g at 1 bar, which is almost 5 times greater than that observed for the original GO12, and 2.4 times the adsorption capacity of the material doped with nitrogen GO12PANI (Figure 5A).

Figure 5B shows the adsorption isotherms of the mixed hydrogels prepared at different GO24/ACF ratios. For comparison, the adsorption isotherms of GO24 and of the hydrogel GO24H have been included. As can be seen in Figure 5B, the hydrothermal treatment to GO24 increases, the CO_2_ adsorption capacity from 0.22 to 0.64 mmol/g at 1 bar, reaching a level close to that obtained with N-doped GO (GO12PANI) without chemical activation. In the CO_2_ adsorption isotherms obtained for the GO/ACF mixed hydrogels, it is observed that the adsorption capacity of the hydrogels increases as the ACF content increases. The hydrogel with the lowest proportion of ACF (GO24FH73) provides a CO_2_ retention capacity of 2.23 mmol/g at 1 bar, exceeding the retention capacity of GO12PANIK, the N-doped composite with the best adsorption capacity. The CO_2_ uptake increases as the GO24/ACF ratio increases, up to 3.59 mmol/g at 1 bar for GO24FH37. However, note that no synergistic effects have been observed between GO24 and ACF, and the retention capacity presented by pure ACF (5.42 mmol/g at 1 bar) has not been exceeded in any mixed hydrogels. This fact can be due to the existence of separated domains in the hydrogels, as was demonstrated by Raman spectra and SEM images. To improve the homogeneity of hydrogels and avoid the existence of two separated domains, we added the surfactant cetylpyridinium chloride to the hydrogel of 50% GO24/ACF ratio. The result shows a slight increase in the retention capacity of GO24FH55CPy (Table 2), but not a synergistic effect. Although a thermodynamic mixture of the two domains could be expected using the surfactant solution, it has not been achieved, as can be seen in Figure S5 in the Supporting Information.

**Figure 6:**
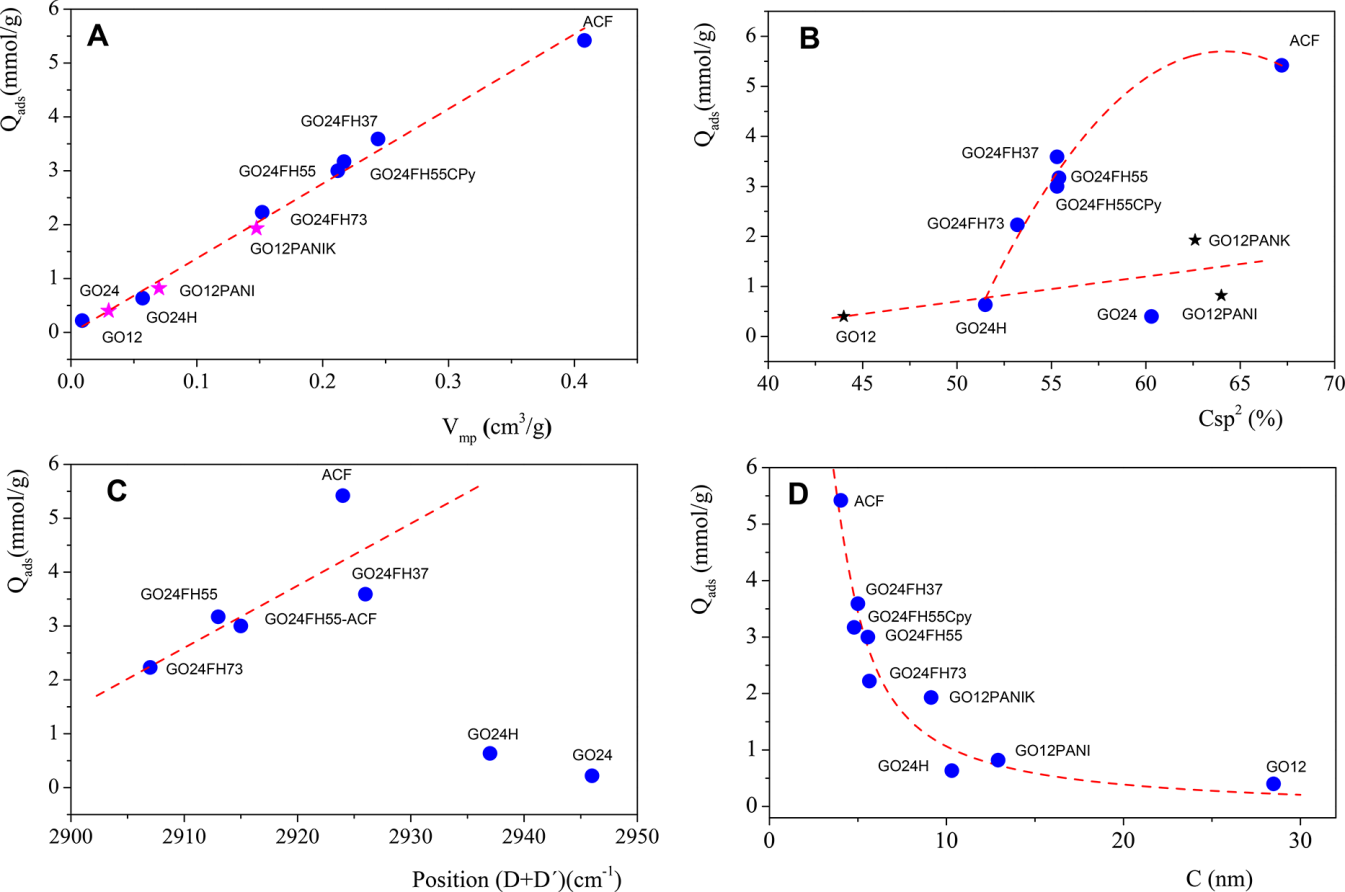
Plot of the CO_2_ uptake at 1 bar against (A) the volume of micropores from the Dubinin–Radushkevich equation, (B) the percentage of C sp^2^ obtained by XPS, (C) the position of the (D+D′) band of the second-order Raman spectra, and (D) the grain size *C* by the XDR (100) peak from the Scherrer equation.

To analyze the influence of the structural properties on the CO_2_ retention capacity, Figure 6 presents some of the evaluated properties with the CO_2_ uptake at 1 bar. In Figure 6A, the CO_2_ uptake at 1 bar is plotted versus the micropore volume. The results show an excellent linear correlation, which agrees very well with previous results.^32^ This fact confirms again that CO_2_ is physisorbed in micropores, and consequently, the increase of the micropore volume results in a more efficient CO_2_ adsorption. It becomes necessary to investigate the structural properties responsible for the increase of micropores in these solids. Therefore, in Figure 6B, we analyze the influence of percentage of C sp^2^ on the CO_2_ uptake. The results in Figure 6B do not show a clear trend between these two parameters. Although the amount of CO_2_ adsorbed in the hybrids that contain ACF increases with the percentage of C sp^2^, it does not occur for the rest of the materials, where the increase in CO_2_ adsorption capacity with the percentage of C sp^2^ is very slight. Similar information has been obtained from the plot of the CO_2_ adsorption capacity against the position of the combination band (D + D′). In Figure 6C, for samples hybridized with ACF, the adsorption capacity increases when the position of the band, related to the percentage of C sp^2^, shifts toward higher wave numbers. It can be concluded that another structural property more related with the structure of the fibers should be responsible for the increase of the micropore volume observed in these samples. With this objective in mind, Figure 6D shows the variation of the CO_2_ uptake with the grain size *C*, obtained by XRD. As can be seen in Figure 6D, the CO_2_ retention capacity decreases when the grain size increases. Since the retention capacity linearly increases with the micropore volume, our results unequivocally demonstrate that the solids with small grain size present a higher micropore volume and consequently, higher CO_2_ retention capacity.

The results obtained lead us to conclude that the CO_2_ retention capacity is strongly conditioned by the presence and proportion of ACF, so that the porosity of the fiber governs the behavior of mixed hydrogels.

Regarding the preparation methodology, we started with two graphene oxides prepared with different oxidation times, and the conclusion obtained is that the oxidation time does not significantly modify the CO_2_ uptake. The hydrothermal method improves the adsorption capacity of GO to 12% of the ACF capacity and drives to a similar adsorption capacity (15%) to that obtained by doping GO with N synthesized by in situ polymerization of aniline. Activation with KOH of the last material represents a substantial increase of the CO_2_ retention capacity, reaching a value close to 36% of the ACF adsorption capacity. However, the introduction of microporous ACF in hydrogels assumes a high increase of the ability of CO_2_ adsorption. Furthermore, this capacity increases with the proportion of ACF, but a synergistic effect was not detected (see Figure S7 in the Supporting Information).

## Conclusions

In this work, different procedures have been used to synthesize GO-based nanomaterials designed to retain CO_2_, with the objective of determining the structural factors that affect the quality of the adsorbent. Our results prove again that micropore volume is a crucial parameter to improve the CO_2_ retention capacity. We also demonstrate that the C sp^2^ is not a determining parameter in the creation of microporosity. From Raman and adsorption results, it appears that the type of defects in the graphenic network does not have a great influence on CO_2_ retention, although more efforts should be made to clarify this subject. However, our results demonstrate a clear dependence of the CO_2_ retention capacity with the grain size *C* obtained by XRD measurements. Thus, when the crystallite size decreases, CO_2_ retention significantly increases. On the other hand, the different methodologies that have been proposed for the preparation of adsorbents, viz., hydrothermal synthesis, doping with N, activation with KOH, and mixing with fibers, involve successive modifications that progressively increase the amorphous character of the materials, decrease the grain size, and therefore, improve the adsorption capacity. We expect that these results help prepare carbon-derived adsorbents with the highest CO_2_ adsorption capacity.

## Supporting Information Available

Details about the structural characterization of GO/PANI composites and GO/ACF hydrogels using XPS, XRD, and micro-Raman spectroscopy (Section S1); adsorption isotherms, details about the micropore volume analysis (Dubinin–Radushkevich model), and N_2_ adsorption isotherms of the GO24/ACF hydrogels (Section S2); comparison between the CO_2_ adsorption capacities at 273 K and 1 bar of the different composites synthesized in the work (Section S3) (PDF)

## Abbreviations

GO12: graphene oxide obtained by oxidation of graphite during 12 h
GO24: graphene oxide obtained by oxidation of graphite during 24 h
GO24H: hydrogel of GO24 obtained by the hydrothermal method at 180 °C.
GO12PANI: nanocomposite obtained by GO12 and polyaniline by polymerization in situ.
GO12PANIK: nanocomposite obtained by GO12 and polyaniline by polymerization in situ activated with KOH and thermally at 550 °C
GO24FH37: hydrogel obtained by GO24 and activated carbon fiber with a weight ratio of 30/70 by the hydrothermal method at 180 °C
GO24FH55: hydrogel formed by GO24 and activated carbon fiber with a weight ratio of 50/50 by the hydrothermal method at 180 °C
GO24FH73: hydrogel formed by GO24 and activated carbon fiber with a weight ratio of 70/30 by the hydrothermal method at 180 °C
GO24FH55CPy: hydrogel formed by GO24 and carbon fiber with a weight ratio of 50/50 in the presence of cetyl piridinium bromide by the hydrothermal method at 180 °C
ACF: activated carbon microporous fiber
