# Supplementary material for: Optimizing the Properties of Hybrids Based on Graphene Oxide for Carbon Dioxide Capture
Source: Ind Eng Chem Res. 2022 Jan 13;61(3):1332–43. doi: 10.1021/acs.iecr.1c02922 (PMC8796650; doi:10.1021/acs.iecr.1c02922)
Supplement: Supplementary file 1 — ie1c02922_si_001.pdf [file ie1c02922_si_001.pdf]

Optimizing the properties of hybrids based on graphene oxide for carbon dioxide capture

Yating Ye[a], L. Vega Martín[a], M. J. Sánchez Montero[a, b, c]; D. López-Díaz[a, d],M.M. Velázquez[a, b, c]; M.D. Merchán[a, b, c*]

[a]*Departamento de Química Física, Facultad de Ciencias Químicas, Universidad de Salamanca, E-37008 Salamanca, Spain*

[b]*Grupo de Nanotecnología, Universidad de Salamanca, E37008 Salamanca, Spain*

[c]*Laboratorio de Nanoelectrónica y Nanomateriales, USAL-NANOLAB, Universidad de Salamanca, E37008 Salamanca, Spain*

[d]*Departamento de Química Analítica, Química Física e Ingeniería Química, Universidad de Alcalá. 28871 Alcalá de Henares, Madrid, Spain.*

* Corresponding author. Tel: +34 670 547 110. Email: mdm@usal.es (M. Dolores Merchán)

SECTION 1. ***Characterization of GO/PANI composites and GO/ACF hydrogels***

XPS Analysis

Table S1. Percentages of different elements obtained from XPS measurements.

**Sample**	**% C**	**%O**	**%N**	**%S**	**%K**	**C/O**	**% Csp^2**	**% Csp^3**
GO12	68	29	0.8	0	0	2.3	44	56
GO12PANI*	87.6	6	6.4	0	0	14.3	64	36
GO12PANIK	84.8	6.8	7.5	0	0.9	12.5	63	37
GO24	41.4	48.7	0	9.9	0	0.9	60.3	39.7
GO24H	86.3	15.1	0	0.6	0	6.6	51.5	48.5
GO24FH73	76.9	21.1	0	2.0	0	3.7	53.2	46.8
GO24FH55	79.6	18.9	0	1.6	0	4.2	55.3	44.7
GOFH55CPy	78.1	19.1	1.1	1.7	0	4.1	55.4	44.6
GPFH37	80.8	17.8	0	1.4	0	4.5	55.3	44.7
ACF	91.1	8.9	0	0	0	10.3	67.2	32.8

(*) Data taken from ref. 1

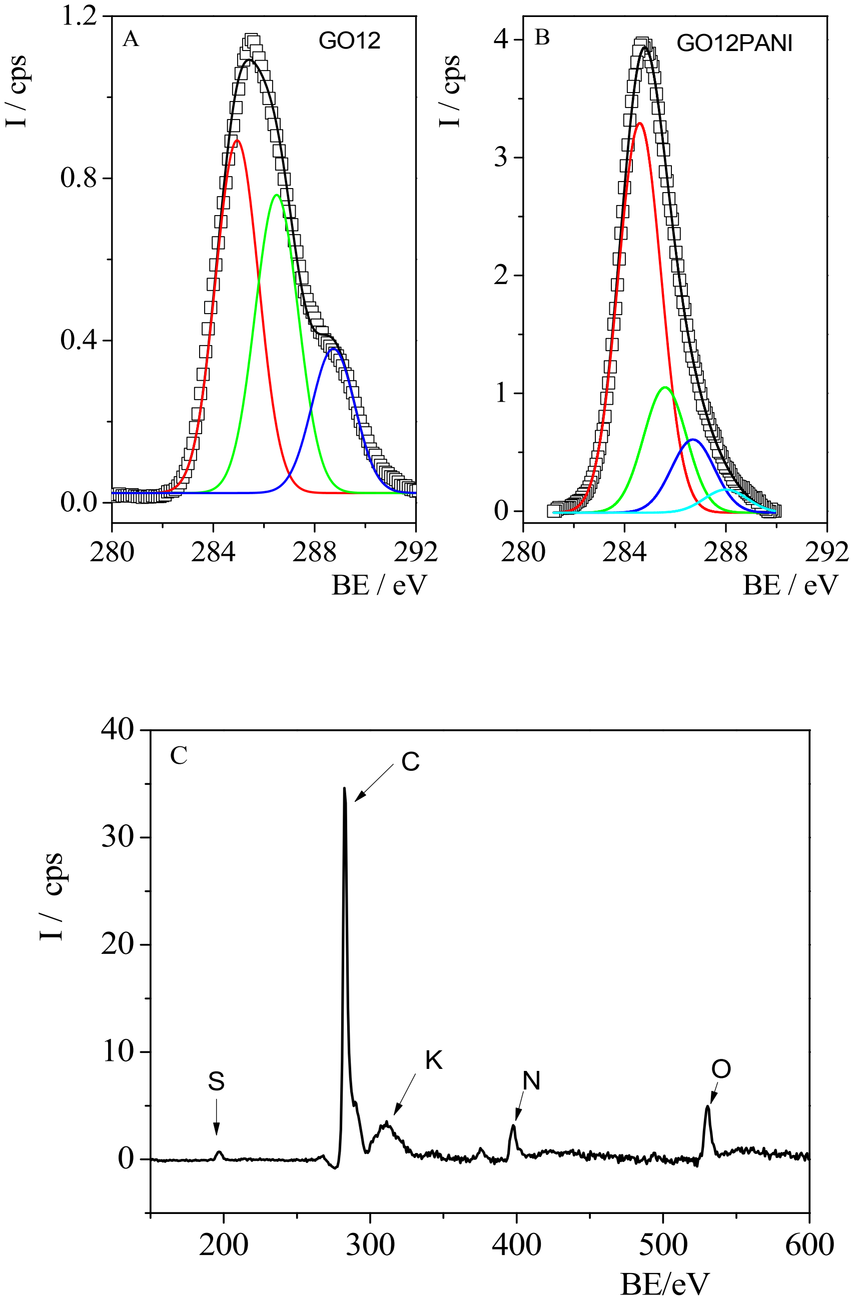

Figure S1: X-ray photoelectron spectra of C1s core level of **A)** GO12 and **B)** GO12PANI. **C)** Survey of XPS of GO12PANIK.

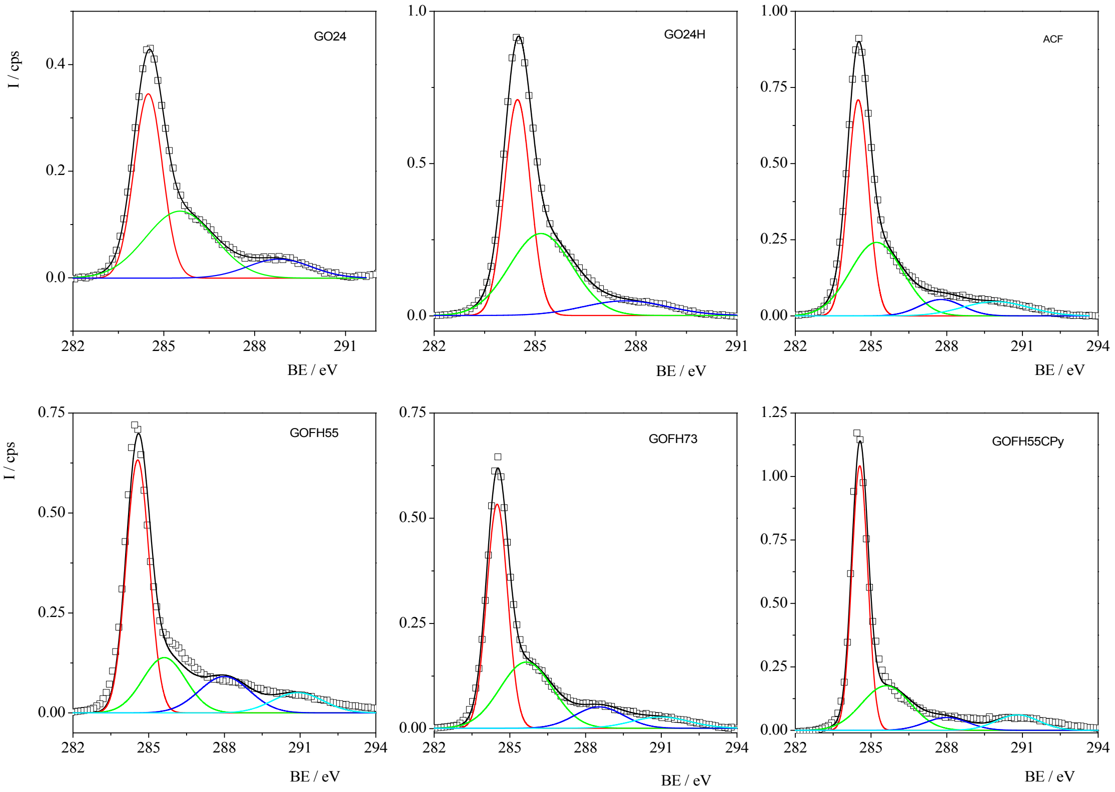

Figure S2: X-ray photoelectron spectra of C1s core level of GO24, GO24H. Activated carbon fibers and hydrothermal composites of GO24 and carbon fibers GO/Carbon Fibers ratio of: 50/50. GOFH55; 70/30. GOFH73 and GO/Carbon fibers ratio of 50/50 in cethyl pyridinium chloride.

XRD Analysis

Table S2. Interlayer distance calculated by the Bragg´s law (002) peak. Analysis of the grain size, C by the XDR (100) peak by Scherrer´s equation: C=0.9 λ/(βcosθ). λ=1.5405 Å; β=half weight width in radians; θ is the diffraction angle. GO24 doesn´t show (100) reflection.

Sample	Bragg´s Law		Scherrer´s equation		
	2θ / º	d / nm	2θ / º	β / º	C / nm
GO12	25.5	0.35	42.23	0.299	28.48
GO12PANI	24.7	0.36	43.05	0.661	12.92
GO12PANIK	25.9	0.34	43.41	0.936	9.14
GO24	24.0	0.37	-	-	-
GO24H	24,6	0.36	42.61	0.83	10.30
GO24FH73	24,5	0.36	42.68	1.51	5.65
GO24FH55	24,5	0.36	42.53	1.53	5.57
GOFH55CPy	24,4	0.36	42.80	1.78	4.79
GOFH37	24,2	0.37	43.56	1.71	5.00
ACF	-	-	43.76	2.12	4.03

Raman Analysis

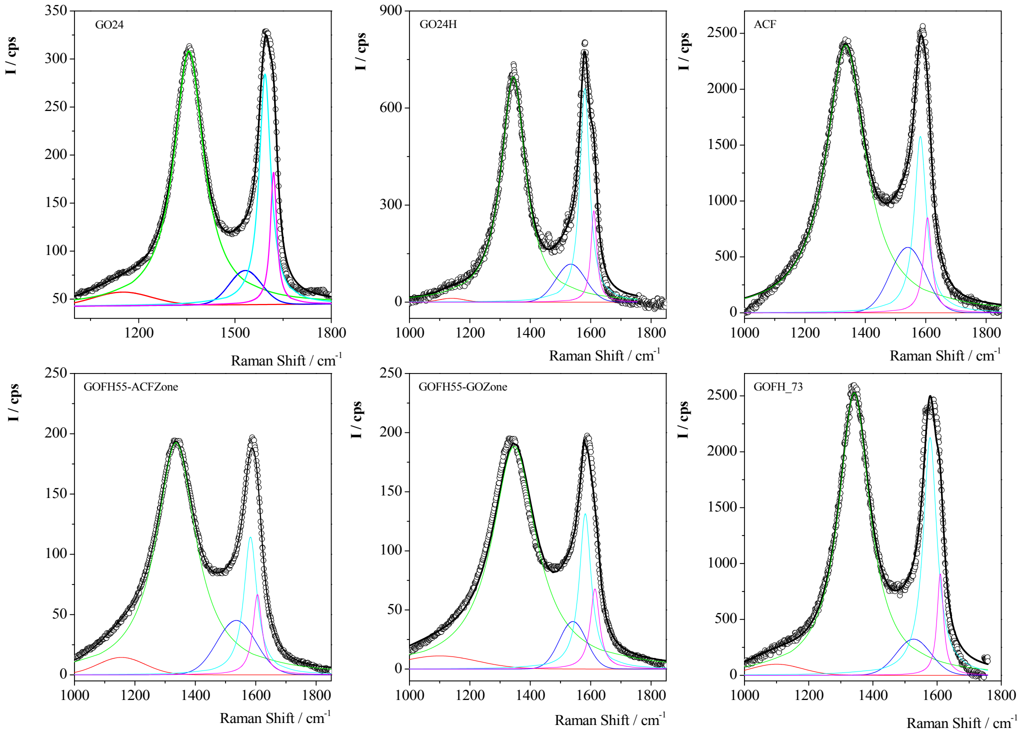

Figure S3: Deconvolution of the first order Raman spectrum of GO24, GO24H, GOFH73, GO24FH55 and Carbon Fibers.

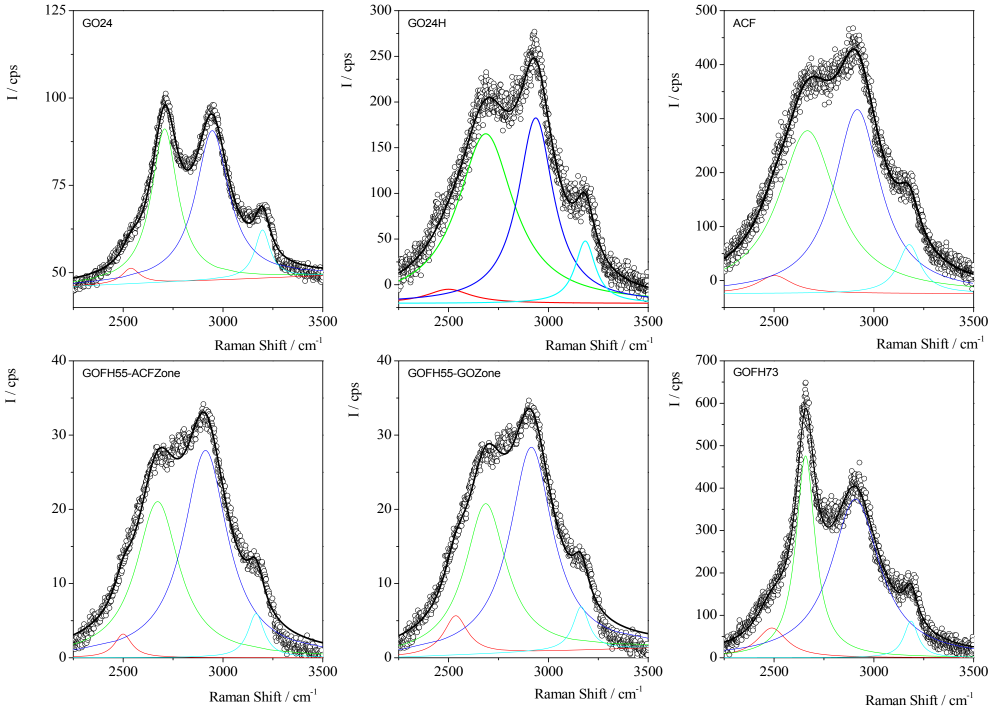

Figure S4: Deconvolution of the second order Raman spectrum of GO24, GO24H, GOFH73, GO24FH55 and Carbon Fibers.

Table S3: Fitting parameters for hydrogels of the first-order Raman spectra

Material	D* (Gauss)			D(Ps-Voight)			D´´(Gauss)			G (Ps-Voight)			D´(Ps-Voight)		
	x_c (cm^{-1})	w(cm^{-1})	A(%)	x_c (cm^{-1})	w(cm^{-1})	A(%)	x_c (cm^{-1})	w(cm^{-1})	A (%)	x_c (cm^{-1})	w(cm^{-1})	A (%)	x_c (cm^{-1})	w(cm^{-1})	A(%)
GO24	1150	213	4	1356	112	61	1533	117	6	1594	44	22	1620	24	7
GO24H	1140	84	1	1344	99	62	1534	98	8	1580	39	23	1610	24	6
GO24FH73	1100	159	3	1342	119	64	1527	108	6	1578	51	23	1610	24	5
GO24FH55-F	1156	156	4	1336	159	67	1536	129	10	1583	53	13	1606	36	5
GO24FH55-GO	1100	249	5	1347	176	70	1541	90	6	1582	47	13	1614	37	5
GO24FH37	1100	265	5	1345	128	64	1530	107	7	1585	51	17	1609	31	6
ACF	1100	52	~0	1335	159	71	1541	136	10	1582	48	14	1606	32	5

Table S4: Relative intensity for Hydrogels of some Raman bands.

Material	$A_{D´}/A_D$	I_D/I_G
GO24	0.11	1.10
GO24H	0.10	1.35
GO24FH73	0.07	1.19
GO24FH55-ACF zone	0.08	1.69
GO24FH55-GO zone	0.14	1.43
GO24FH37	0.09	1.45
ACF	0.07	1.52

Table S5: Fitting parameters for different samples of the second-order Raman spectra

Material	G* (Lorentz)			2D(Lorentz)			D+D´(Lorentz)			2D´ (Lorentz)		
	x_c (cm^{-1})	w(cm^{-1})	A (%)	x_c (cm^{-1})	w(cm^{-1})	A (%)	x_c (cm^{-1})	w(cm^{-1})	A (%)	x_c (cm^{-1})	w(cm^{-1})	A (%)
GO24	2538	109	3	2708	160	40	2946	201	50	3198	94	7
GO24H	2500	271	4	2686	342	53	2937	215	36	3185	124	7
GO24FH73	2489	183	7	2658	117	30	2907	293	59	3186	87	4
GO24FH55-F	2500	105	2	2673	261	39	2913	268	54	3166	100	4
GO24FH55-GO	2536	145	6	2686	252	37	2915	260	53	3.164	98	4
GO24FH37	2563	165	6	2693	225	33	2926	242	54	3178	109	6
Carbon Fibers	2525	74	1	2670	337	53	2924	259	43	3172	81	3

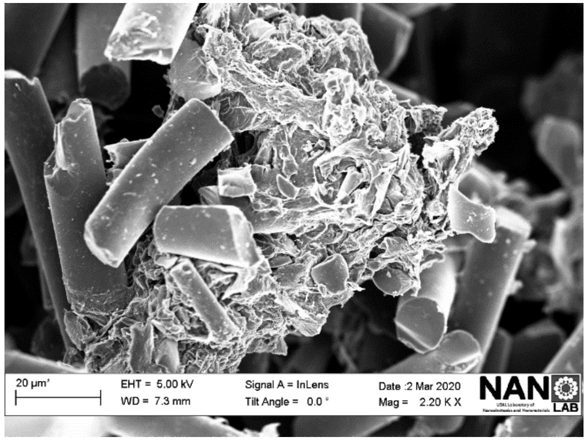

Figure S5: SEM image of GOFH55CPy

SECTION 2. *Adsorption isotherms*

Micropore Volume analysis (Dubinin-Radushkevich model)

The micropore volume was calculated attending to Dubinin-Radushkevich model, eq. S1.

$$\log V = \log V_0 - \left[\frac{RT}{E_0}\right]^2 \left[log\left(\frac{p^0}{p}\right)\right]^2 \qquad \text{(S1)}$$

In eq. S1, V represents the volume of CO_2 adsorbed at (cm^3/g STP) at T and p^o/p. V_o is the micropore capacity (cm^3/g STP) and E_o represents the characteristic energy of adsorption. V is calculated trough V=Δn M/ρ where Δn is CO_2 adsorbed in mol/g, M is the molar mass and ρ is the density of CO_2 at a given temperature. The volume of micropore is expressed as the volume of CO_2 condensed in micropores, obtained by the conversion of the experimental data with the density conversion factor, D (cm^3 liquid/cm^3 STP).

N_2 *adsorption isotherms*

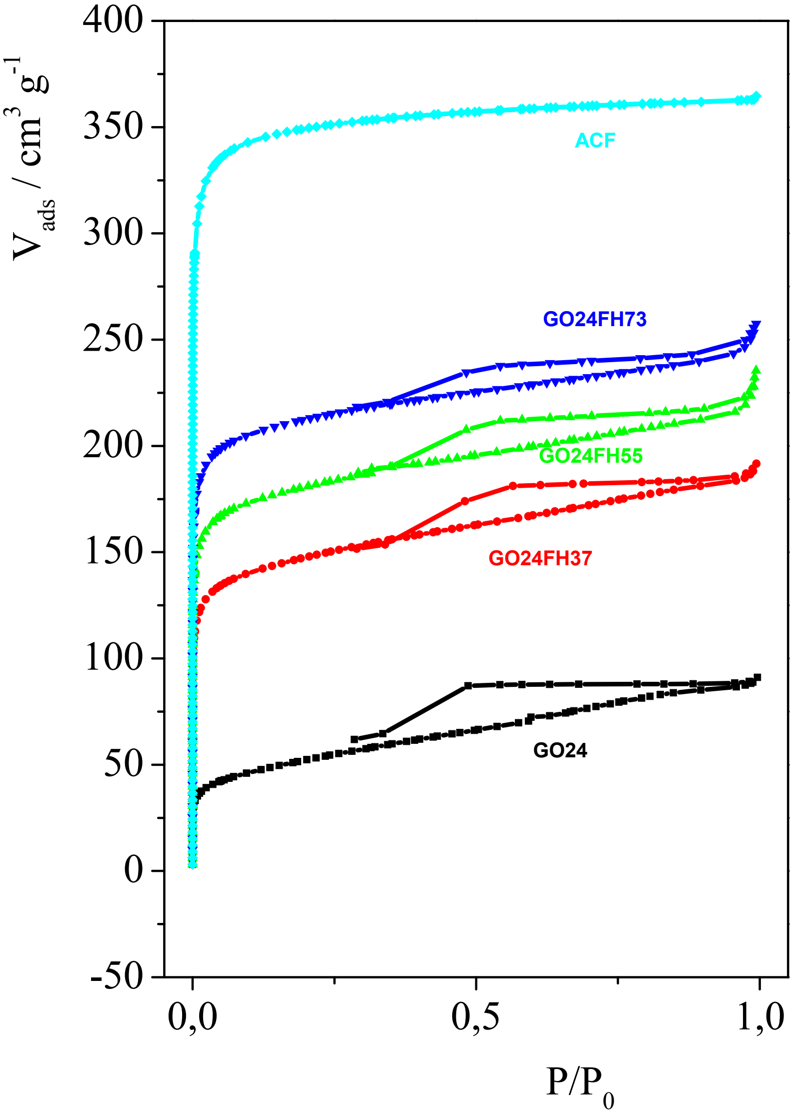

Figure S6: N_2 adsorption-desorption isotherm of the GO24/ACF hydrogels, and ACF at 77K.

Adsorption of CO_2

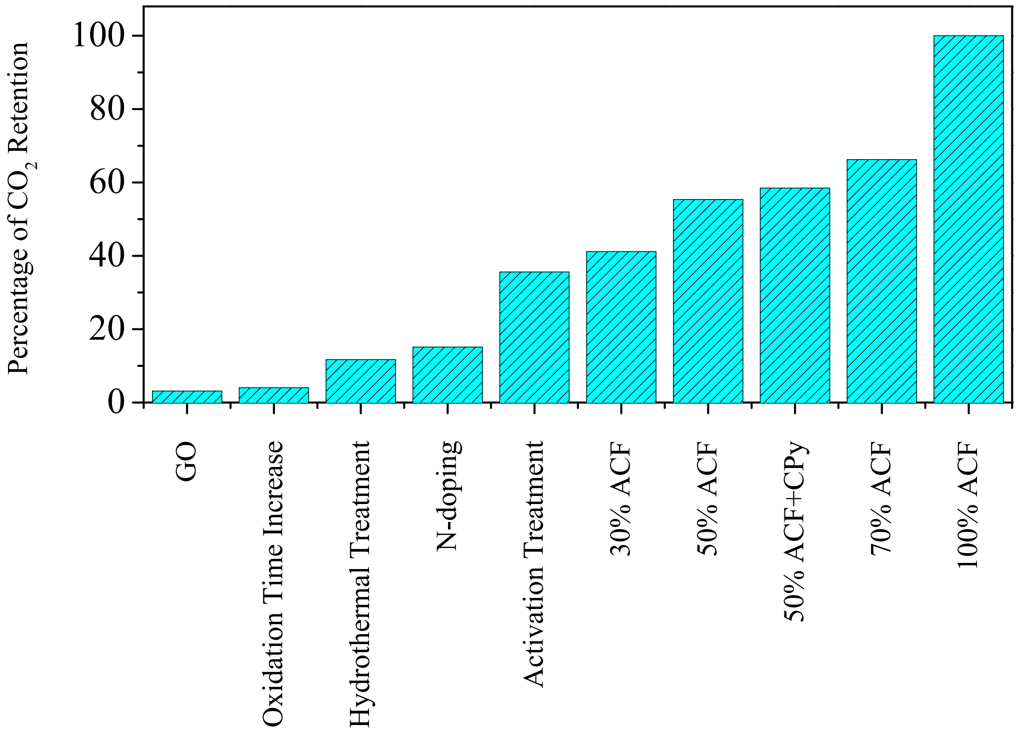

Figure S7: Comparison of the different strategies employed to improve the CO_2 retention relative to the CO_2 uptake of the ACF at 1 bar.

Acknowledgment

The work was financed by European Regional Development Fund, ERDF, and Junta de Castilla y León (SA121P20 and 2020/00325/001). Authors also acknowledge USAL-NANOLAB for Raman facility. XPS measurements were performed in Servicio de espectroscopia de fotoelectrones de Rayos X de la Universidad de Málaga.

Authors acknowledge financial support from Junta de Castilla y León (2020/00325/001).
Dr. Merchán wishes to thank the time she worked with Prof. Fierro. From him she learned to love the hours in the laboratory and the need for total dedication to obtain the best results. She values and maintains Prof. Fierro's model of meticulousness, patience, rigor and wisdom.

Supporting Information

SECTION 1: Details about the structural characterization of GO/PANI composites and GO/ACF hydrogels using XPS, XRD and Micro-Raman spectroscopy.
SECTION 2: Adsorption isotherms. Details about the Micropore Volume analysis (Dubinin-Radushkevich model) and N_2 adsorption isotherms of the GO24/ACF hydrogels.
SECTION 3: Comparison between the CO_2 adsorption capacities at 273K and 1 bar of the different composites synthesized in the work.

TOC

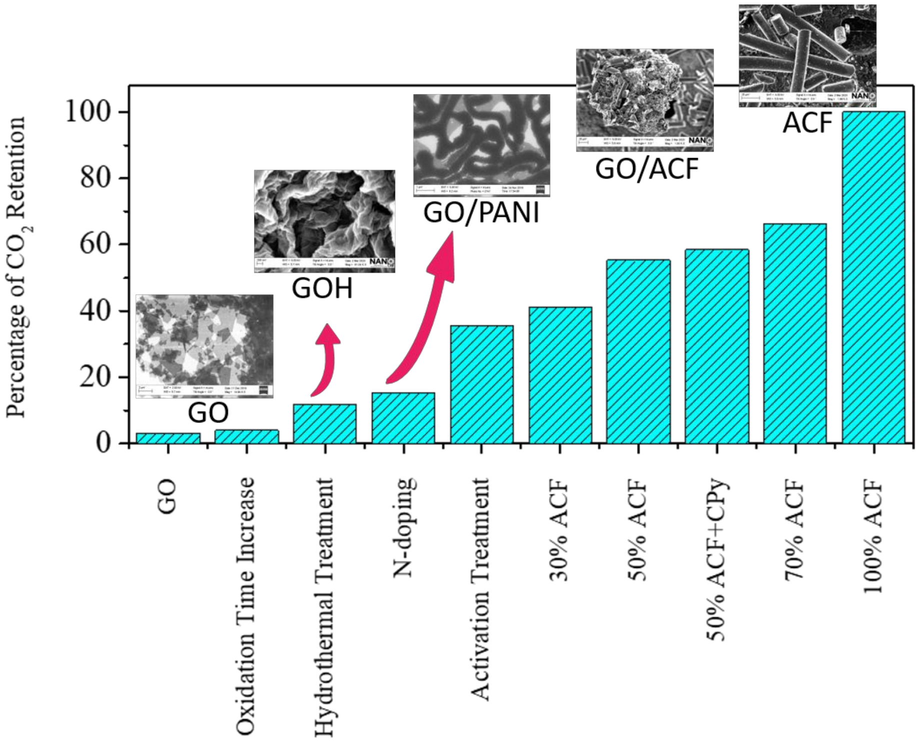

Percentage of CO_2 Retention
0
20
40
60
80
100
GO
GOH
GO/PANI
GO/ACF
ACF
GO
Oxidation Time Increase
Hydrothermal Treatment
N-doping
Activation Treatment
30% ACF
50% ACF
50% ACF+CPy
70% ACF
100% ACF
